# Whooping Cough

**Published:** 1881-01

**Authors:** 


### WHOOPING COUGH.

CALLED PERTUSSIS by physicians, is said to be owing to the presence of bacteria under the root of the tongue. The cough is so urgent that the breath becomes exhausted, and, in the effort to draw in the needed air, the top of the windpipe seems to nearly close, causing a whooping sound. The old plan was to let the disease run its course; being careful that the symptoms were not aggravated by cold.

Certain medicines seem to act favorably in some cases, and in others to have no noticeable effect. The following have been recommended :

The fluid extract of hyoscyamus, from 1 to 4 drops, depending on the age of patient. A blister to back of neck. Frictions to the spine twice a day, with onion juice. Beat a fresh egg in a pint of vinegar, and add half a pound of loaf sugar ; take two tablespoonfuls every four hours.

THE BEST TREATMENT

for the cure of whooping cough that has yet been discovered undoubtedly is to expose the patients to the vapors arising from the purifying boxes in gas-works. But it is not always convenient for the patient to visit these places; and it has been found to answer just as well to procure some of the liquid hydro-carbon that is always found at the bottom of the purifying boxes, and vaporize it in a metal dish in the closed room of the little patient. It almost always affords immediate relief, and the whooping will not return for hours. This liquid can be obtained at any gas-works at little or no cost, and no expense need be incurred for vaporizers. A tablespoonful or more may be evaporated at a time, and repeated once a day or oftener. A large iron spoon makes a good evaporating dish. It may be held over the flame of a lamp, or a coal shovel may be heated and the liquid poured into it and allowed to evaporate in the room of the patient. This treatment would probably be beneficial in throat and lung troubles. It is safe, convenient and inexpensive.

---

THE greatest man is he who chooses right with the most invincible resolution ; who resists the sorest temptations from within and without ; who bears the heaviest burdens cheerfully ; who is the calmest in storms, and the most fearless under menaces and frowns.

